# Thomas Rose

**Published:** 1829-02

**Authors:** 


We have still another melancholy record to place upon our pages, which will be deeply felt both by the profession and society in general. Thomas Rose, Esq. aged forty-five, A.M. surgeon of St. George's Hospital, and late surgeon of the Coldstream Regiment of Foot Guards, departed this life at his house in Park place, on the 21st of January. The heavy domestic affliction with which Mr Rose had been recently visited, laid the foundation for the disease which speedily removed him from all the turmoil of this world. He was known to be most ardently attached to his domestic circle; and the unexpected loss of three children, who fell victims to hooping-cough at Boulogne, and who were all (we believe) buried in the same grave on the same day, was too severe a blow to be borne by the ordinary philosophy of human nature. Mr. Rose's health sank rapidly under the poignant anguish of his mind, and it soon became too evident that he never would recover the shock.

Eulogies upon the dead are so indiscriminately, and therefore so injudiciously lavished, that but little reliance may sometimes be placed on the praises which truth demands. In bestowing the warmest panegyric upon the professional and private character of Mr. Rose, we shall not, however, be accused of exaggerating his merit. By all who knew him he was acknowledged to be able as a surgeon, and most scrupulously honourable and kindhearted as a man.

